# New insights into enzymatic hydrolysis of heterogeneous cellulose by using carbohydrate-binding module 3 containing GFP and carbohydrate-binding module 17 containing CFP

**DOI:** 10.1186/1754-6834-7-24

**Published:** 2014-02-19

**Authors:** Shuhong Gao, Chun You, Scott Renneckar, Jie Bao, Yi-Heng Percival Zhang

**Affiliations:** 1 School of Biotechnology, State Key Laboratory of Bioreactor Engineering, East China University of Science and Technology, Shanghai 200237, China; 2 Biological Systems Engineering Department, Virginia Tech, 304-A Seitz Hall, Blacksburg, VA 24061, USA; 3 Sustainable Biomaterials Department, Virginia Tech, 230 Cheatham Hall, Blacksburg, VA 24061, USA; 4 Cell-Free Bioinnovations Inc., 2200 Kraft Drive, Suite 1200B, Blacksburg, VA 24060, USA; 5 Gate Fuels Inc., Blacksburg, VA 24060, USA

**Keywords:** Amorphous cellulose, Enzymatic hydrolysis of heterogeneous cellulose, Carbohydrate-binding module, Crystalline cellulose, Mono-cherry fluorescent protein

## Abstract

**Background:**

The in-depth understanding of the enzymatic hydrolysis of cellulose with heterogeneous morphology (that is, crystalline versus amorphous) may help develop better cellulase cocktail mixtures and biomass pretreatment, wherein cost-effective release of soluble sugars from solid cellulosic materials remains the largest obstacle to the economic viability of second generation biorefineries.

**Results:**

In addition to the previously developed non-hydrolytic fusion protein, GC3, containing a green fluorescent protein (GFP) and a family 3 carbohydrate-binding module (CBM3) that can bind both surfaces of amorphous and crystalline celluloses, we developed a new protein probe, CC17, which contained a mono-cherry fluorescent protein (CFP) and a family 17 carbohydrate-binding module (CBM17) that can bind only amorphous cellulose surfaces. Via these two probes, the surface accessibilities of amorphous and crystalline celluloses were determined quantitatively. Our results for the enzymatic hydrolysis of microcrystalline cellulose (Avicel) suggested that: 1) easily accessible amorphous cellulose on the surface of Avicel is preferentially hydrolyzed at the very early period of hydrolysis (that is, several minutes with a cellulose conversion of 2.8%); 2) further hydrolysis of Avicel is a typical layer-by-layer mechanism, that is, amorphous and crystalline cellulose regions were hydrolyzed simultaneously; and 3) most amorphous cellulose within the interior of the Avicel particles cannot be accessed by cellulase.

**Conclusions:**

The crystallinity index (CrI), reflecting a mass-average (three-dimensional) cellulose characteristic, did not represent the key substrate surface (two-dimensional) characteristic related to enzymatic hydrolysis.

## Introduction

Cellulose, the primary component of plant cell walls, is the most abundant renewable biopolymer on earth. The biodegradation of cellulose is essential to the complete carbon cycle and will be vital to next generation biorefineries that will produce biofuels, value-added biochemical, and even food [1]. Enzymatic hydrolysis of this heterogeneous cellulose is a complicated biological process requiring synergetic actions among endoglucanase, exoglucanase, and β-glucosidase [2]. An in-depth understanding of enzymatic cellulose hydrolysis mechanisms related to cellulose characteristics could help develop more effective biomass pretreatment methods, more active cellulase, and more easily degraded genetically-modified plants. These impacts would further enable the cost-effective release of fermentative soluble sugars from non-food biomass [3].

Numerous biomass substrate characteristics are believed to influence the enzymatic hydrolysis rate and digestibility, such as substrate accessibility, crystallinity, degree of polymerization (DP), particle size, pore volume, lignin content, and so on [4-6]. Most times, cellulose accessibility including external and internal areas is closely associated with several other substrate characteristics, such as particle size, pore volume, and crystallinity [7]. Cellulose accessibility can be measured by numerous technologies, such as nitrogen adsorption-based Brunauer–Emmett–Teller, water vapor sorption, alkali swelling, an exchange of H to D atoms with D_2_O, size exclusion chromatography, small angle X-ray scattering, microscopy, Simon dyes, active cellulase adsorption, and a family 3 carbohydrate-binding module (CBM3)-containing fluorescent protein [7-9]. It is important to note that the dried cellulosic samples have a completely different supramolecular structure and substrate reactivity from hydrated cellulosic samples [7,10]; additionally, many experiments use cellulose-surface probing molecules (for example, dinitrogen, D_2_O) that are small relative to the enzymes and a true probe should have similar size to cellulases. Several fusion proteins containing a fluorescent protein and a carbohydrate-binding module (CBM) have been used to qualitatively visualize the polysaccharide recognition on cellulose [11,12] but not quantitatively. Specifically, a quantitative measurement for determining cellulose accessibility to cellulase (CAC) was established by using a non-hydrolytic fusion protein containing a green fluorescent protein (GFP) and CBM3, which was cloned from the *cipA* gene in *Clostridium thermocellum*, called GC3 [8]. Via this technology, it was found that increasing CAC was strongly related to enhanced cellulose digestibility according to both experimental data [6,7] and prediction from the functionally-based kinetic models [13,14]. However, GC3 can bind both surfaces of amorphous cellulose and crystalline cellulose fragments, but its binding cannot distinguish the heterogeneous surfaces of cellulosic materials.

A CBM is a polypeptide module found in carbohydrate-active enzymes that can specifically bind to different carbohydrates. CBMs have been classified into 68 different families based on amino acid sequences and molecular structures (http://www.cazy.org/Carbohydrate-Binding-Modules.html). Based on the three-dimensional structure and functional similarity, CBMs are classified into three types: surface binding (type A); glycan chain binding (type B); and small sugar binding (type C) [15]. CBM3, belonging to type A, can bind both surfaces of crystalline and amorphous celluloses [8,12]. CBM17 is a family 17 carbohydrate-binding module, belonging to type B [16,17]. This module contains clefts that accommodate single polysaccharide chains that are associated with amorphous cellulose areas [16,18,19]. Therefore, it can specifically bind to the surface of amorphous cellulose but not to that of crystalline cellulose [16,17].

Microcrystalline cellulose (Avicel) is a typical model cellulosic substrate for the study of enzymatic cellulose hydrolysis [14,20]. Although it is made through acid hydrolysis that can remove most amorphous cellulose and all hemicellulose, Avicel is a morphologically heterogeneous solid substrate containing amorphous cellulose and crystalline cellulose regions [10,20]. Enzymatic hydrolysis of Avicel or other heterogeneous cellulosic materials, mediated by the non-complexed enzyme mixture, is a peeling or layer-by-layer process [21-23]. The new ends of cellulose chains that are cleaved by endoglucanase can only be hydrolyzed by the adsorbed exoglucanase after the endoglucanase moves away from the local area [21,24]. Traditionally, it was hypothesized that the amorphous cellulose regions in heterogeneous Avicel were hydrolyzed prior to the hydrolysis of crystalline cellulose [2]. However, the slight changes or even no changes in crystallinity index (CrI) values during the hydrolysis process cannot justify the severalfold reduction in the hydrolysis rate for the whole hydrolysis process [8,25-28].

To understand the in-depth mechanism of enzymatic hydrolysis of the heterogeneous cellulose morphology of Avicel and test the widely believed hypothesis that amorphous cellulose regions of Avicel are hydrolyzed prior to crystalline cellulose, we designed a new non-hydrolytic fusion protein, called CC17. This new probe contains a mono-cherry (mCherry) fluorescent protein and CBM17, which can specifically bind to the surface of amorphous cellulose, but not to that of crystalline cellulose [18]. The adsorption of both fusion proteins containing a CBM and a fluorescent protein can be quantitatively measured based on its fluorescent signal with very high sensitivity compared to other protein assays, such as UV adsorption and Bradford protein assay. By applying this tool, we quantitatively studied the changes of accessibilities of amorphous cellulose and crystalline cellulose regions during the enzymatic hydrolysis of Avicel.

## Results and discussion

### CC17 production and purification

**Figure 1:**
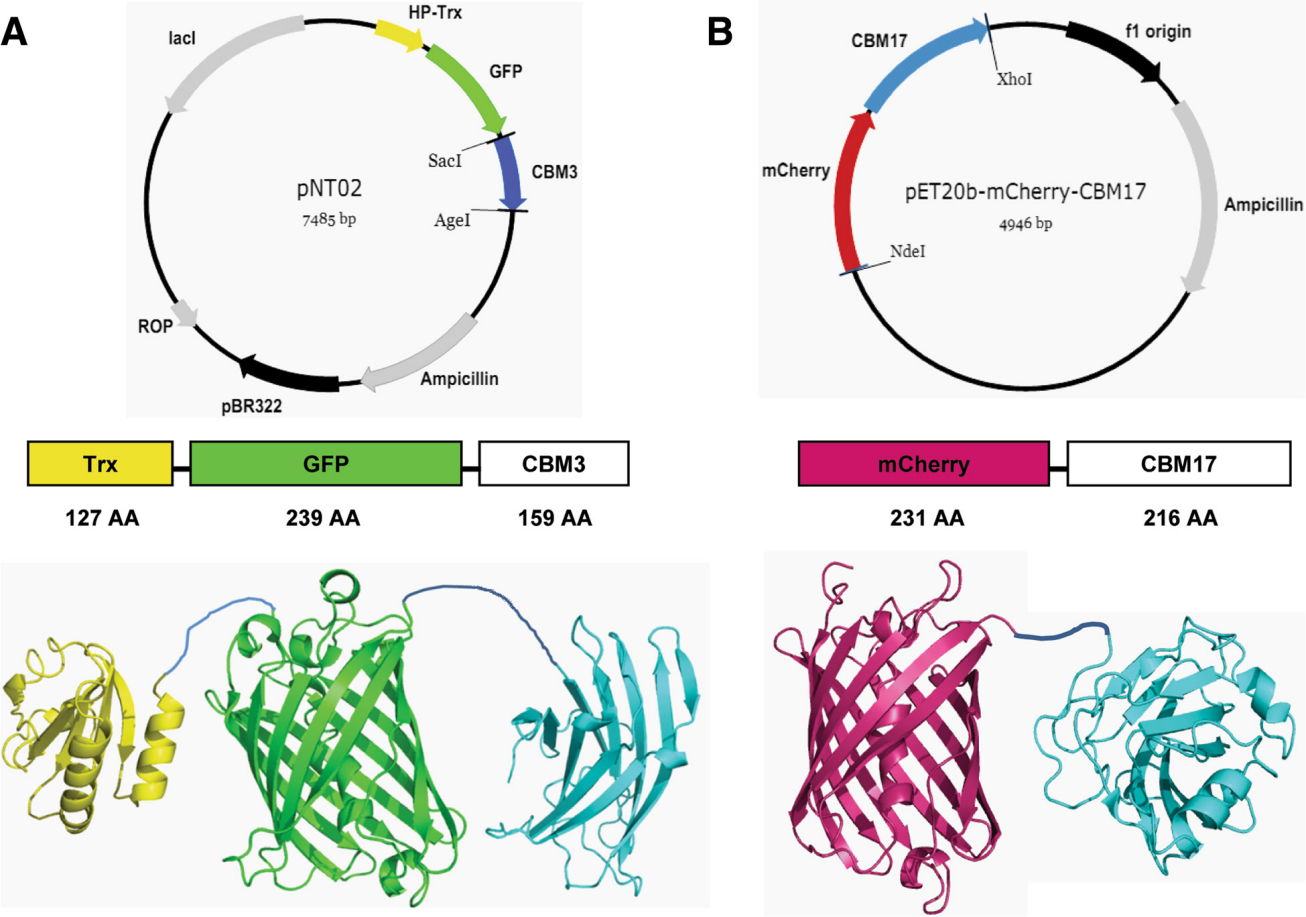
**Plasmid maps, schemes, and molecular structures. (A)** GC3 and **(B)** CC17. CC17, mono-cherry fluorescent protein linked to a family 17 carbohydrate-binding module; GC3, green fluorescent protein linked to a family 3 carbohydrate-binding module.

**Figure 2:**
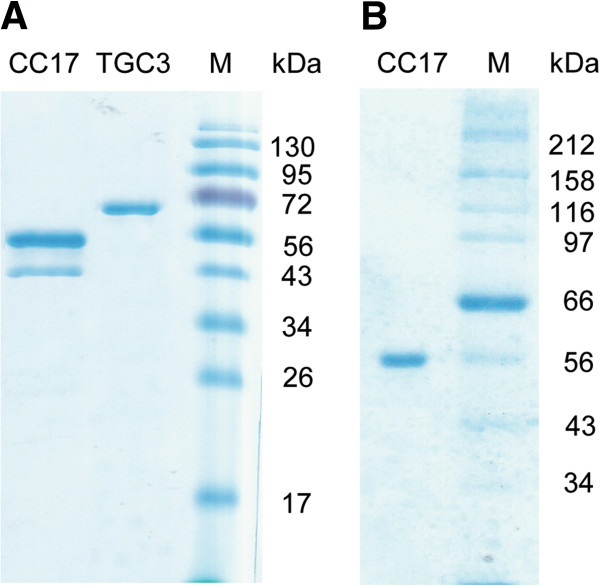
**SDS-PAGE analysis. (A)** Purified GC3 and CC17 in a typical denaturation buffer. **(B)** Purified CC17 in a modified PAGE, where SDS concentration was increased to 1% in gel and 0.3% in the running buffer. CC17, mono-cherry fluorescent protein linked to a family 17 carbohydrate-binding module; GC3, green fluorescent protein linked to a family 3 carbohydrate-binding module.

Because CBM17 can specifically bind amorphous cellulose, a recombinant fusion protein CC17 was designed to contain a mono-cherry fluorescent protein (CFP) linked with a CBM17 to monitor adsorption with high sensitivity. The recombinant CC17 was expressed in *Escherichia coli* BL21 (DE3) harboring plasmid (Figure 1B). Its molecular weight (52.1 kD) is similar to the size of most cellulases. Another recombinant GC3 (62.9 kD) containing thioredoxin, a GFP, and a CBM3 was used to determine the total cellulose accessibility to cellulase (TCAC) [8]. Both of the recombinant proteins, CC17 and GC3, were expressed very well in *E. coli* BL21 (DE3) strains. The purified proteins of CC17 and GC3 were examined to confirm whether they were homogenous by SDS-PAGE (Figure 2A). The purified CC17 protein exhibited two bands, one with an expected size and one with a smaller size, under a typical SDS-PAGE condition (Figure 2A), leading to a speculation of possible degradation of CC17 during its expression and purification. After a series of diagnostic experiments that involved changing the linker’s amino acid composition (data not shown), two bands of CC17 in typical SDS-PAGE were attributed to the incomplete denaturation of CC17, resulting in two forms of CC17 that had different movement rates in SDS-PAGE. By increasing the SDS concentration tenfold in the protein denaturation buffer, CC17 exhibited a single band and its molecular weight was approximately 54 kD, similar to the predicted one based on its deduced amino acid sequence (Figure 2B).

### Quantitative determination of cellulose accessibility to cellulase (CAC)

The previous study of GC3 adsorption on bacterial microcrystalline cellulose (BMCC) suggested that one molecule of GC3 occupied 21.2 cellobiose lattices on the 110 face of crystalline cellulose, revealing the alpha value of 21.2 for GC3 [8]. Note, although the exact binding site of a CBM3 is much smaller than 21.2 cellobiose lattices [29], the whole GC3 occupies more space due to the crowding effect for the large-sized adsorbed fluorescent protein on the surface of cellulose. As a result, the cross-sectional area of GC3 (Figure 1A) is significantly larger than that of 21.2 × 0.53 × 1.04 nm. Because GFP is a dimer protein, it was thought that the adsorbed GC3 formed a dimer on the surface of BMCC, resulting in an underestimated alpha value by twofold. Therefore, in reality, one GC3 monomer was thought to occupy 42.4 cellobiose lattices. Surprisingly, Levine and his co-workers used the revised alpha value of 42 in their mechanistic model without any explanation [30].

**Figure 3:**
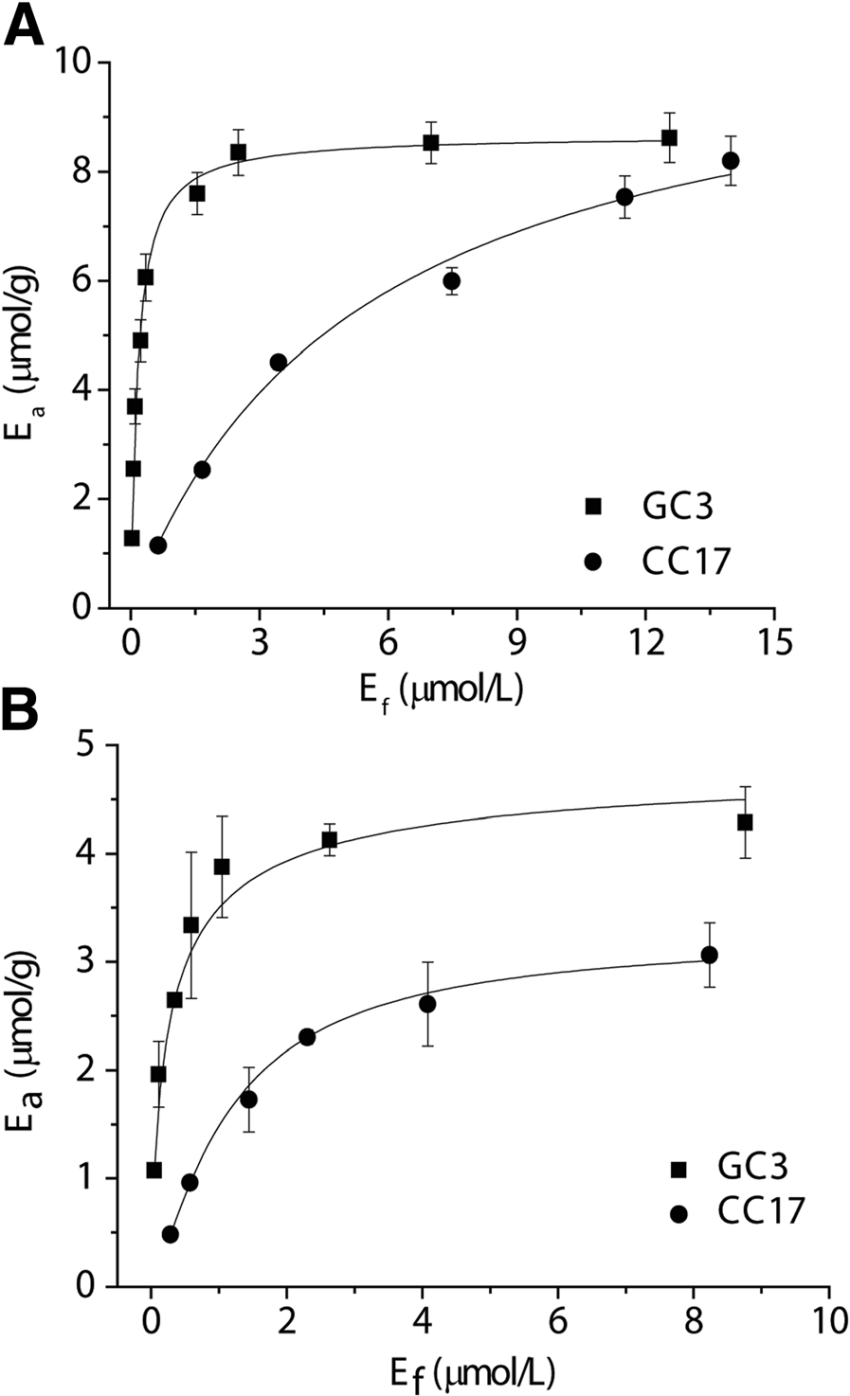
**Adsorptions of GC3 and CC17 proteins on 0.2 mg/mL of RAC in a 100 mM HEPES buffer (pH 7.5) containing 2 M NaCl at room temperature. (A)** Single-component adsorption of GC3 and CC17. **(B)** Two-component adsorption of GC3 in the presence of 4.48 μmol/L CC17 and of CC17 in the presence of 1.54 μmol/L GC3. GC3 (■) and CC17 (●). All curves were fit with the Langmuir isotherms. CC17, mono-cherry fluorescent protein linked to a family 17 carbohydrate-binding module; GC3, green fluorescent protein linked to a family 3 carbohydrate-binding module; RAC, regenerated amorphous cellulose.

To quantitatively determine the beta value for CC17 on amorphous cellulose (that is, the number of cellobiose lattices that was occupied by one molecule of CC17), we measured the TCAC based on regenerated amorphous cellulose (RAC), which was made by the dissolution in ice-cooled concentrated phosphoric acid followed by water precipitation. The CrI of freeze-dried RAC was zero, suggesting that there was no crystalline cellulose after cellulose dissolution and regeneration [10]. The adsorptions of both CC17 and GC3 followed the Langmuir equations (Figure 3A). The maximum protein adsorption capacities were 8.64 ± 0.15 μmol of GC3 and 11.28 ± 0.26 μmol of CC17 per gram of RAC, respectively (Table 1). Therefore, the total cellulose accessibility of RAC was 120.4 ± 2.1 m^2^/g RAC (see Equation 1 in the Methods section); this value related physically to one molecule of CC17 occupying 32.3 cellobiose lattices because the total accessibility of RAC can be bound by CC17. This beta value for CC17 was reasonable, compared to the alpha value for GC3 mentioned above, because one dimeric GC3 was proportionally larger compared to one monomeric CC17 (Figure 1).

**Table 1:** Maximum adsorption capacities of the GC3 and CC17 on RAC and the determination of the beta value for CC17 that specifically binds to the amorphous cellulose surface

**Protein**	**A**_**max**_	**TCAC**^a^	**Alpha/beta value**
	**(μmol/g RAC)**	**(m**^**2**^**/g RAC)**	**(cellobiose lattice/protein)**
GC3	8.64 ± 0.15	120	42.4
CC17	11.28 ± 0.26	120	32.5

^a^TCAC = aCAC, because RAC is 100% amorphous cellulose [10]. aCAC, amorphous cellulose accessibility to cellulase; CC17, mono-cherry fluorescent protein linked to a family 17 carbohydrate-binding module; GC3, green fluorescent protein linked to a family 3 carbohydrate-binding module; RAC, regenerated amorphous cellulose; TCAC, total cellulose accessibility to cellulase.

In addition to the single-component (GC3 or CC17) adsorption (Figure 3A), the two-component adsorption of GC3 and CC17 was examined on RAC. Clearly, due to the presence of 4.48 μmol/L CC17 protein, the maximum binding capacity of GC3 was decreased to 4.87 ± 0.22 (Figure 3B) from 8.64 ± 0.15 μmol per gram of RAC (Figure 3A), suggesting that both CBM3 and CBM17 competitively bind the surface of amorphous cellulose. Similarly, in the presence of 1.54 μmol of GC3 per L, the maximum binding capacity of CC17 decreased to 3.26 ± 0.18 from 11.28 ± 0.26 μmol per L. The total accessibility of RAC measured by using either GC3 or CC17 alone was approximately 365 cellobiose lattices, very close to the sum of the accessibilities measured by GC3 in the presence of CC17 or vice versa. This result suggests that CBM3 and CBM17 can both bind the same amorphous cellulose surface regions of RAC.

### Enzymatic hydrolysis of Avicel

**Figure 4:**
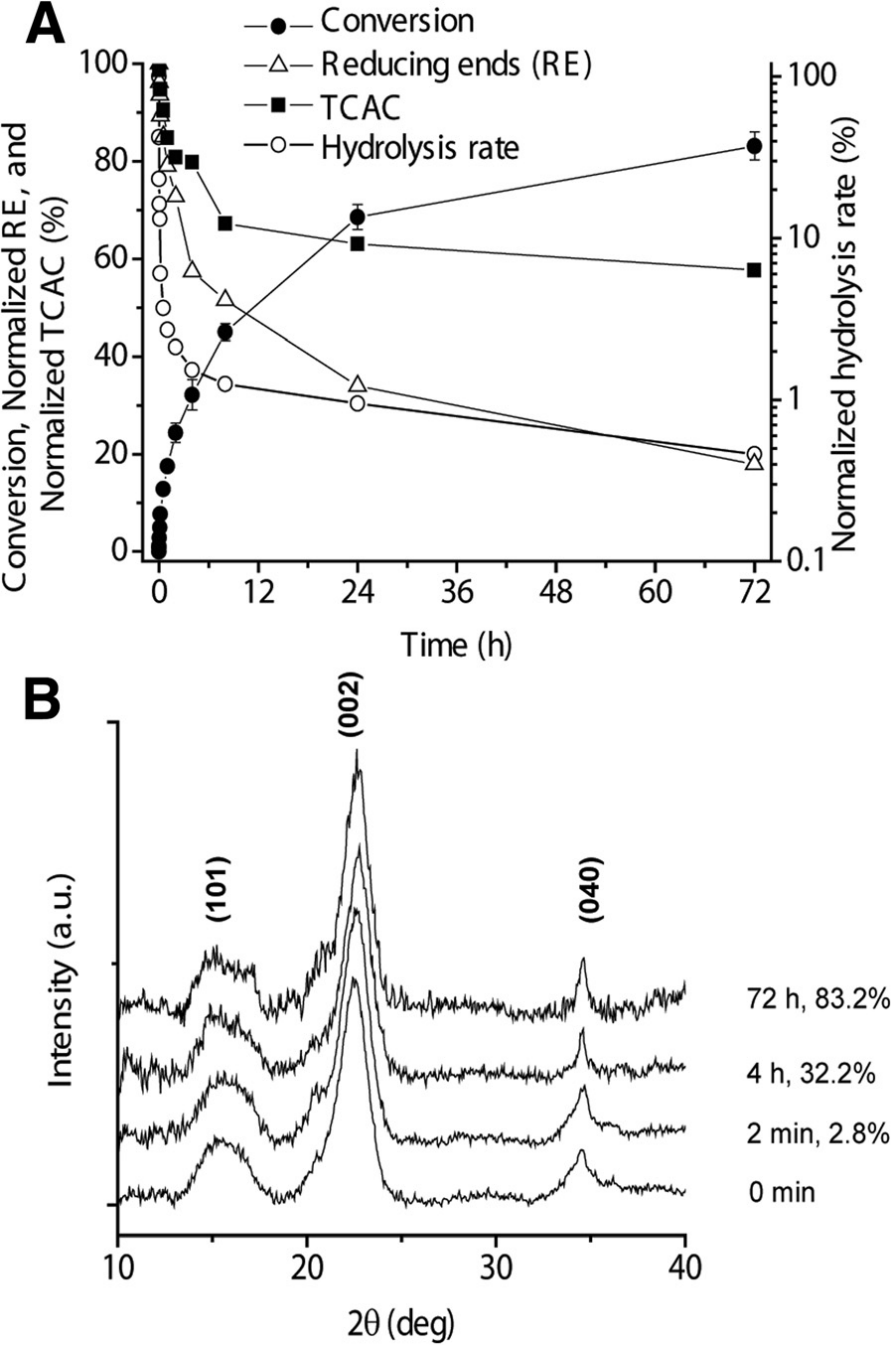
**Profiles of enzymatic hydrolysis of Avicel (10 g/L) by 15 FPU of cellulase and 30 units of β-glucosidase per gram of cellulose in a 50 mM sodium citrate buffer (pH 4.8) at 50°C. (A)** Changes of cellulose conversion (●), hydrolysis rate (○), reducing ends (△), and TCAC (■). **(B)** X-ray diffraction spectra of intact and freeze-dried samples with different hydrolytic conversions. FPU, filter paper unit; TCAC, total cellulose accessibility to cellulase.

**Figure 5:**
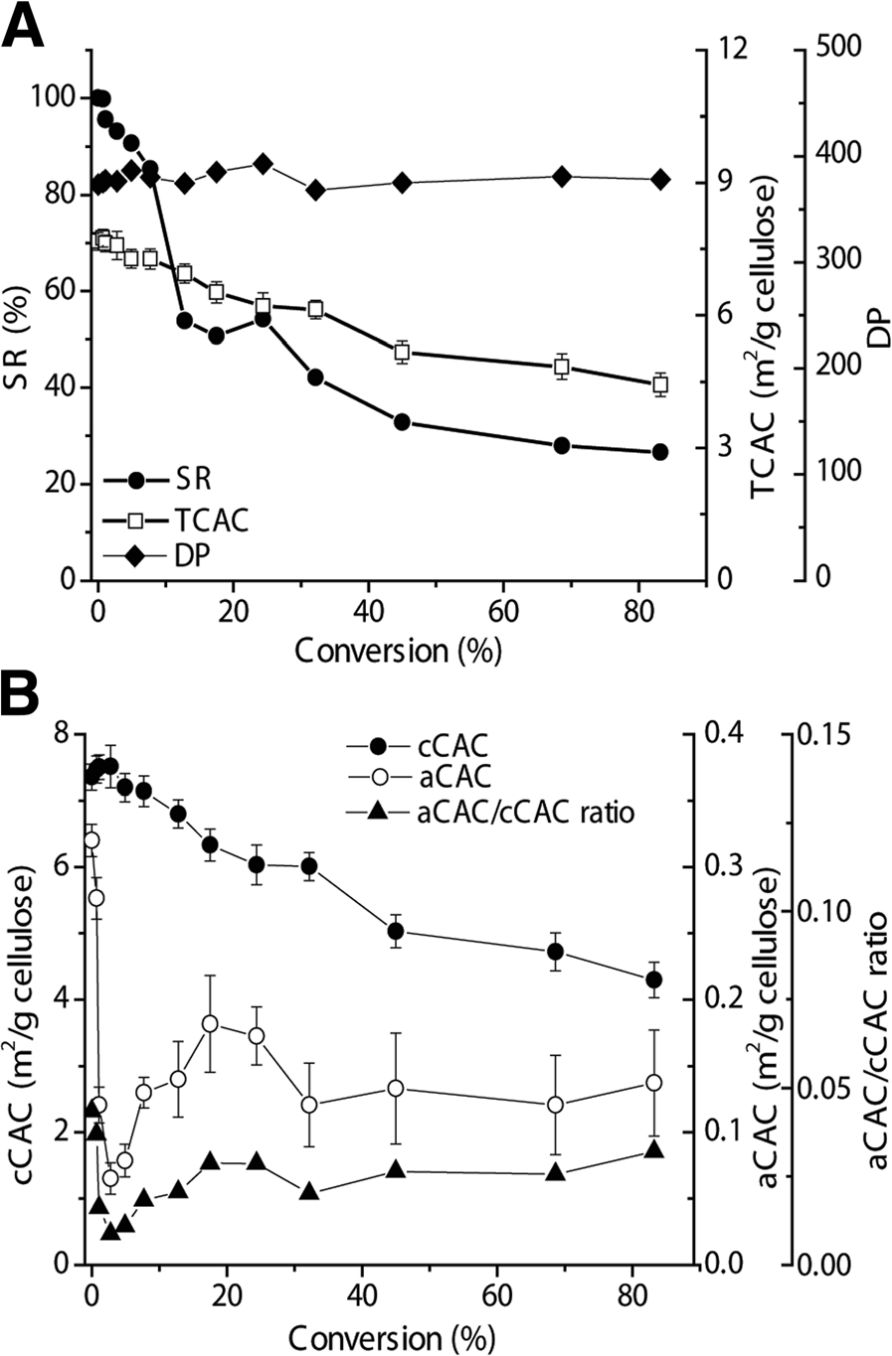
**Profiles of enzymatic hydrolysis of Avicel as a function of substrate conversion. (A)** Changes of SR (●), TCAC (□), and DP (◆). **(B)** Changes of aCAC (○), cCAC (●), and ratio of aCAC to cCAC (▲). aCAC, amorphous cellulose accessibility to cellulase; cCAC, crystalline cellulose accessibility to cellulase; DP, degree of polymerization; SR, substrate reactivity; TCAC, total cellulose accessibility to cellulase.

Enzymatic hydrolysis of a model heterogeneous microcrystalline cellulose (Avicel) mediated by the fungal *Trichoderma* cellulase was conducted with an enzyme loading of 15 filter paper units (FPUs) of cellulase and 30 units of β-glucosidase per gram of Avicel at 50°C (Figure 4). Avicel was hydrolyzed rapidly in the first 4 hours with a cellulose conversion of 32.3% and then slowly, achieving a final conversion of 82.3% at hour 72 (Figure 4A). The normalized hydrolysis rates of Avicel decreased rapidly by 84% at the first 2 minutes with 2.8% conversion; this was followed by a decrease to less than 4% of the initial hydrolysis rate in the next 30 minutes, where the conversion was 12.8%. For the period from hour 4 to 72, it slowly decreased to 1.5% and 0.4% of the initial rate, respectively (Figure 5A). Such a drastic decrease in enzymatic hydrolysis rates could be attributed to the combined effects of several factors: 1) cellulose consumption; 2) enzyme deactivation; 3) product inhibition; and 4) the loss in substrate reactivity. With regard to cellulose consumption, total CAC decreased from 7.67 ± 0.20 to 6.13 ± 0.21 m^2^/g cellulose at hour 4, and to 4.43 ± 0.27 m^2^/g cellulose at hour 72, and reducing ends of solid cellulose decreased from 235 to 135 μM at hour 4 to 42 μM at hour 72 (Figure 4A). This data suggests reduced surface area for enzyme adsorption of enzyme binding domains and reduced chain ends for hydrolysis by the exoglucanase. With regard to enzyme deactivation, it was known that commercial cellulase was relatively stable under experimental conditions (50°C for several days). With regard to product inhibition, the addition of 30 units of β-glucosidase resulted in very low cellobiose levels in the supernatant (data not shown), suggesting low bulk product inhibition. However, a recent discovery that fast substrate channeling occurs along the reactive surface of cellulose relative to adjacent microorganisms implies that product inhibition in the boundary layer on the surface of cellulose may often be underestimated [31].

The CrI of cellulose is widely regarded as a key substrate characteristic that affects enzymatic cellulose hydrolysis rates based on a wide observation that amorphous cellulose can be hydrolyzed much faster than crystalline cellulose [20]. Many techniques, such as X-ray diffraction (XRD) [32], cross-polarization magic angle spinning ^13^C nuclear magnetic resonance (CPMAS ^13^C NMR) [33,34], and Fourier transform infrared spectroscopy (FTIR) [35], have been employed to determine CrI values. CrI values can vary greatly depending on measurement techniques, calculation approaches, and sample drying conditions [10,34]. Meanwhile, CrI represents an average reading of the entire cellulose (three-dimensional) property rather than an average cellulose surface (two-dimensional) property. In this study, the changes of CrI values at different conversions were minimal (Figure 4B). These results suggested that the CrI was not a sensitive substrate indicator of substrate characteristic relating to declined enzymatic hydrolysis rates over conversion.

Substrate reactivity (SR) of cellulose hydrolyzed by cellulase is an intrinsic characteristic of the solid substrate that can be measured based on a hydrolysis rate of a fixed concentration of cellulose (10 g/L) hydrolyzed by a fixed amount of freshly added cellulase. Therefore, SR changes are independent of substrate consumption, enzyme denaturation, and product inhibition. The study of the residual cellulose during the enzymatic hydrolysis could help understand the root cause of declined hydrolysis rates over conversion (Figure 5). The normalized SR declined rapidly to approximately 50% when the substrate conversion reached approximately 20%, and then declined slowly to approximately 30% of the initial rate (Figure 5A). In contrast, the TCAC determined based on the binding capacity of GC3 gradually decreased from 7.67 ± 0.20 to 4.43 ± 0.27 m^2^/g cellulose. During the entire hydrolysis process, the DP of residual cellulose was nearly constant (Figure 5A), suggesting the typical layer-by-layer hydrolysis mechanism. If enzymes were able to penetrate throughout the heterogeneous substrate, the core chains of the cellulose would have a lower DP, as occurs to the hydrolysis of amorphous cellulose [24].

To further investigate changes of CACs, including amorphous CAC (aCAC) and crystalline CAC (cCAC), the surface of amorphous cellulose was determined based on the maximum capacity of CC17 (Figure 5B). aCAC decreased rapidly from 0.320 ± 0.012 to 0.065 ± 0.012 m^2^/g cellulose at the conversion of 2.8%, then slowly increased to approximately 0.17 m^2^/g cellulose at the conversion of 24.4%, and then leveled off around 0.13 m^2^/g cellulose until the conversion reached 83.2%. The cCAC value remained nearly constant at the first conversion of 2.8%, being 7.40 m^2^/g cellulose, and then decreased gradually to 6.03 ± 0.32 and 4.29 ± 0.31 m^2^/g cellulose at the conversion of 24.4% and 83.2%, respectively. As a result, the ratio of aCAC to cCAC decreased rapidly from 0.043 ± 0.001 to 0.0087 ± 0.001 at the very early beginning of hydrolysis, and then increased slowly to 0.032 ± 0.007 at the conversion of 83.2%.

New insights about enzymatic hydrolysis of Avicel were reported by using two CBM systems. First, aCAC in Avicel accounted for a small fraction of the entire CAC (5%) although amorphous cellulose contents determined were approximately 30 to 45% by XRD or ^13^C NMR [10]. This surprising difference between CrI and aCAC/TCAC suggested that most amorphous cellulose fragments were embedded inside the cellulose fibers, which cannot be accessed by large-sized protein molecules (for example, CC17 and cellulases). This structure may occur as microfibrils coalesce together during acid hydrolysis, as the size of isolated nanocystalline cellulose is always reported to be larger than native cellulose microfibrils in wood. The result of nearly no change in DP during the entire conversion (Figure 5A) also suggested the layer-by-layer hydrolysis; both amorphous and crystalline surface are peeled nearly simultaneously by the synergetic action of endoglucanase and exoglucanase, which was partially supported by no change of CrI (Figure 4B). Second, a small fraction of amorphous cellulose fragments within intact Avicel was highly accessible by CC17 or cellulase. The initial fast decrease in cellulose hydrolysis rates during the first several minutes (that is, before the conversion of 2.8%) was attributed to preferential hydrolysis of this kind of amorphous cellulose. A similar observation pertaining to rapid decreases in hydrolysis rates within a short time was reported previously [36]. Our study suggested that this decrease in reaction rate was attributed to the fast consumption of the accessible amorphous cellulose region, which was measured by the adsorption of CC17 (Figure 5B). Third, the increased aCAC to cCAC ratio after the initial hydrolysis period could be attributed to amorphogenesis mediated by cellulases or other non-hydrolytic proteins [5,9].

The use of CrI representing digestibility of cellulosic materials may be a misleading concept because: 1) it represents a mass-average property of cellulose rather than a surface-average property which was strongly correlated with enzymatic hydrolysis; and 2) CrI values could vary greatly depending on measurement techniques, calculation approaches, and sample drying conditions. Furthermore, our data pertaining to the changes of cCAC and aCAC clearly suggested that during the whole hydrolysis period a significant fraction of amorphous cellulose was hydrolyzed simultaneously with crystalline cellulose rather than preferential hydrolysis of amorphous cellulose as hypothesized long before. As a result, there were no significant changes in CrI value, supported by previous results [25-28]. In interpreting crystallinity data, and indeed data for all cellulose physical properties, care must be taken to distinguish correlation from cause and effect. For example, several biomass treatments that not only decrease CrI also increase surface area, and it has been suggested that the increased hydrolysis rates observed with substrates arising from such treatments may be due to increasing adsorptive capacity rather than changes in CrI [37,38]. In summary, we concluded that accessibility of cellulose to cellulase was far more important than CrI in determining the hydrolysis rate.

## Conclusions

In conclusion, a non-hydrolytic fusion protein containing a mono-CFP and a CBM17 was used to quantitatively determine the amorphous cellulose surface area. Our results suggested that: 1) most amorphous cellulose inside Avicel particles cannot be accessed by cellulase; 2) easily accessible amorphous cellulose on the surface of Avicel is preferentially hydrolyzed at the very early period of hydrolysis (that is, several minutes with a cellulose conversion of 2.8%); and 3) further hydrolysis of Avicel is a layer-by-layer hydrolysis process, that is, amorphous and crystalline celluloses were hydrolyzed simultaneously.

## Methods

### Chemicals and strains

All chemicals were reagent grade, purchased from Sigma-Aldrich (St Louis, MO, USA) and Fisher Scientific (Pittsburg, PA, USA), unless otherwise noted. Microcrystalline cellulose, Avicel PH105 (20 μm), was obtained from FMC (Philadelphia, PA, USA). The *Trichoderma* cellulase (Novozym® 50013) and β-glucosidase (Novozyme® 50010) were donated by Novozymes North America (Franklinton, NC, USA). They had activities of 84 FPU per mL and 270 units of β-glucosidase per mL, respectively. RAC was prepared through Avicel dissolution in concentrated phosphoric acid, followed by regeneration in water as described elsewhere [39]. *E. coli* TOP10 from Invitrogen (Carlsbad, CA, USA) was used as a host cell for all DNA manipulations. The Invitrogen *E. coli* BL21 Star (DE3) was used for recombinant protein expression. The Luria-Bertani (LB) medium was used for all *E. coli* growth with 100 μg/mL final concentration of ampicillin when necessary.

### Construction of plasmid

The 706-bp DNA sequence encoding mCherry fluorescent protein was amplified with a pair of primers (IF: TTAAC TTTAA GAAGGAGATA TACAT ATGGT GAGCA AGGGC GAGGA GGATA; IR: CAGTTCATTA TCTGC CCACA GCTTA TCAGA ACCTG GCTTG) using the NEB Phusion polymerase based on plasmid pmCherry from Clontech Laboratories Inc. (Mountain View, CA, USA). The linear pET20b vector backbone containing the gene of a CBM17 was amplified with a pair of primers (VF: CAAGC CAGGT TCTGA TAAGCTGTGG GCAGA TAATG AACTG; VR: TATCC TCCTC GCCCT TGCTC ACCATATGTA TATCT CCTTC TTAAA GTTAA) using the NEB Phusion polymerase based on plasmid pET20b from Novagen (Darmstadt, Germany). The insertion DNA and vector backbone were assembled into DNA multimers by prolonged overlap extension PCR as described elsewhere [40]. The PCR product DNA multimers were transferred to *E. coli* TOP10, yielding plasmid pET20b-mCherry-CBM17 (Figure 1B).

### Preparation of recombinant GC3 and CC17

The recombinant proteins were produced by *E. coli* BL21 harboring the protein expression plasmid. The GC3 fusion protein containing a GFP and CBM3 (Figure 1A) was produced and purified as described elsewhere [6]. Briefly, after isopropyl-β-D-thiogalactopyranoside (IPTG) induction, the GC3 was adsorbed by RAC and then desorbed with ethylene glycol (EG). EG was then removed using membrane dialysis in a 10 mM HEPES buffer (pH 7.5), and the GC3 solution was freeze-dried overnight. The CC17 fusion protein was produced by *E. coli* BL21 Star (DE3) harboring pET20b-mCherry-CBM17 plasmid. In a 1-L flask containing 200 mL of the LB medium, *E. coli* BL21 Star (DE3) was cultured at 37°C. The inducer IPTG (0.1 mM) was added until the absorbency reached 0.6 to 0.8, and then the culture temperature was decreased to 18°C overnight. The cell pellets after centrifugation were suspended in 50 mL of 100 mM HEPES buffer (pH 7.5), and then lysed by the Fisher Scientific sonic dismembrator (Model 500) at a 60% maximum strength for 90 seconds. The cell lysate was centrifuged at 14,000 *g* for 20 minutes. The CC17 protein with a His tag was purified using the Nickel resin column (Bio-Rad, Hercules, CA, USA). The bound protein of CC17 on the resin was eluted by 150 mM imidazole. Imidazole was removed by dialysis in a 10 mM HEPES buffer (pH 7.5) and the CC17 solution was freeze-dried overnight.

### Enzymatic hydrolysis of Avicel

Enzymatic cellulose hydrolysis was conducted in a 1-L flask containing 400 mL of 10 g Avicel/L in 50 mM citrate buffer (pH 4.8) with a rotary rate of 200 rpm and 50°C. The enzyme loadings were 15 FPU of cellulase and 30 units of β-glucosidase per gram of Avicel. The hydrolysate samples were withdrawn and stopped by mixing with 10 M NaOH at a ratio of 20 μL alkali per mL of the hydrolysis slurry [8]. After centrifugation, the soluble sugars in the supernatant were measured by the phenol–sulfuric acid method [24]. The alkalinized solid cellulose pellets were suspended into 1% SDS (final concentration) solution and incubated at 80°C for 15 minutes. After centrifugation, the pellets were suspended and washed three times by both 75% (v/v) ethanol and distilled water [8]. The complete removal of adsorbed cellulase from the surface of cellulose was confirmed by the Lowry assay [41]. The residual cellulose samples were suspended to a 10 g cellulose/L in a 50 mM citrate buffer (pH 4.8) for the assay of SR and a 10 or 50 g/L cellulose in a 100 mM HEPES buffer, 2 M NaCl (pH 7.5) for the adsorption of GC3 or CC17, respectively. The concentration of total glucose equivalent in solid cellulose was measured by the phenol–sulfuric acid method [24]. The relationship between soluble sugars (g/L) versus hydrolysis time (hour) was fitted by CurveExpert (version 1.38). Hydrolysis rates at various times were calculated at the time t + 10 minutes minus sugar produced at the time t. After careful removal of the adsorbed cellulase on the surface of cellulose (see the description above), characteristics of the remaining cellulosic pellets, that is, SR, DP, and CAC, were measured. SR was determined at the conditions: 10 g cellulose/L in the 50 mM citrate buffer (pH 4.8), enzyme loadings of 15 FPU of cellulase and 30 units of β-glucosidase per gram of cellulose, and 50°C. Initial hydrolysis rate at the first 10 minutes based on the released total soluble sugar (glucose equivalent) was used to represent SR.

### Single-component adsorption of GC3 and CC17 and calculation of cellulase accessibilities

The adsorption of GC3 and CC17 on cellulose was conducted in 200 μL of the solution in a 100 mM HEPES buffer (pH 7.5) containing 2 M NaCl at room temperature. The concentration of Avicel was 10 g/L and 50 g/L for the GC3 and CC17 adsorption, respectively. After adsorption equilibrium was achieved (approximately 30 minutes), followed by centrifugation, the free protein molar concentrations of GC3 and CC17 were measured based on their fluorescence readings by the BioTek multi-detection microplate reader (Winooski, VT, USA). The GC3 and CC17 had the excitation of 485 and 590 nm and the emission of 528 and 645 nm, respectively. The extinction coefficients of GC3 and CC17 were 53,890 and 34,260 M^-1^ cm^-1^, respectively. The concentration of proteins was determined by reference to their respective standard curves.

The TCAC in terms of m^2^ per gram cellulose was determined on the basis of the maximum adsorption capacity $\left(A_{\max}^{\mathrm{GC}3}\right)$ of the GC3 protein based on the Langmuir isotherm [6,8]:

(1) $$\mathrm{TCAC}=a\;*\;A_{\text{max}}^{\mathrm{GC}3}\;N_{A}\;*A_{G2}$$

where *α* was the number of cellobiose lattices occupied by GC3 (that is, *α* = 42.4), $\left(A_{\max}^{\mathrm{GC}3}\right)$ = the maximum GC3 adsorption capacity (mol of GC3/g cellulose), *N*_*A*_ = Avogadro’s constant (6.023 × 10^23^ molecules/mol), and *A*_*G2*_ = area of the cellobiose lattice in the 110 face (0.53 × 1.04 nm = 5.512 × 10^-19^ m^2^).

aCAC in terms of m^2^ per gram cellulose was determined based on the maximum adsorption capacity $\left(A_{\max}^{\mathrm{CC}17}\right)$ of the CC17 protein:

(2) $$\mathrm{aCAC}=\beta *A_{\text{max}}^{\mathrm{CC}17}*N_{A}*A_{G2}$$

where *β* was the number of cellobiose lattices occupied by CC17 (that is, *β* = 32.5) and $\left(A_{\max}^{\mathrm{CC}17}\right)$ = the maximum CC17 adsorption capacity (mol of CC17/g cellulose).

cCAC in terms of m^2^ per gram cellulose was calculated as TCAC minus aCAC.

### Two-component adsorption on RAC

Two-component adsorption of GC3 and CC17 on RAC was examined on 0.2 g/L RAC in a 100 mM HEPES buffer (pH 7.5) containing 2 M NaCl at room temperature. When CC17 was 4.48 μmol/L (that is, approximately 40% of its maximum adsorption capacity), a GC3 concentration was adjusted from 0.25 to 10 μmol/L, a mixture of GC3 and CC17 was mixed with 0.2 g/L RAC. After 1 hour of adsorption, the non-bound GC3 in the supernatant was measured based on its fluorescence. Another two-component adsorption experiment was conducted when GC3 concentration was fixed to be 1.54 μmol/L GC3 (that is, approximately 70% of its maximum adsorption capacity) and CC17 concentration was adjusted from 0.3 to 10 μmol/L. When adsorption reached equilibrium at room temperature, the non-bound GC was measured based on its fluorescence.

### Other assays

The purity of purified proteins was examined by 12% SDS-PAGE. The number-average DP of cellulose was calculated by the ratio of glucosyl monomer concentration (determined by the phenol–sulfuric acid method) divided by the reducing-end concentration (determined by the modified bicinchoninic acid (BCA) method) [24]. X-ray diffractograms of all freeze-dried samples were measured using a Bruker D8 Discover X-ray diffractometer (Madison, WI, USA) with the scanning rate of 4°/min, ranging from 10° to 50° [10].

## Abbreviations

13C NMR: ^13^C Nuclear magnetic resonance; aCAC: Amorphous cellulose accessibility to cellulase; BCA: Bicinchoninic acid; BMCC: Bacterial microcrystalline cellulose; CAC: Cellulose accessibility to cellulase; CBM: Carbohydrate-binding module; CBM3: Family 3 carbohydrate-binding module; CBM17: Family 17 carbohydrate-binding module; CC17: mono-cherry fluorescent protein linked to a family 17 carbohydrate-binding module; cCAC: Crystalline cellulose accessibility to cellulase; CFP: Cherry fluorescent protein; CPMAS: Cross-polarization magic angle spinning; CrI: Crystallinity index; DP: Degree of polymerization; EG: Ethylene glycol; FPU: Filter paper unit; FTIR: Fourier transform infrared spectroscopy; GC3: Green fluorescent protein linked to a family 3 carbohydrate-binding module; GFP: Green fluorescent protein; IPTG: Isopropyl-β-D-thiogalactopyranoside; LB: Luria-Bertani; mCherry: Mono-cherry; PCR: Polymerase chain reaction; RAC: Regenerated amorphous cellulose; SR: Substrate reactivity; TCAC: Total cellulose accessibility to cellulase; XRD: X-ray diffraction.

## Competing interests

The authors declare that they have no competing interests.

## Authors’ contributions

SG carried out the protein purification, protein adsorption, enzymatic hydrolysis, and substrate characterization, and drafted the manuscript. CY carried out experiments including the construction of plasmid for CC17 expression, interpreted CC17 denaturation, and drafted the manuscript. SR helped to measure CrI, interpreted CrI data, and revised the manuscript. JB helped to design the experiment and write the draft manuscript. YPZ conceived of the study, participated in its design and coordination, and helped to draft and revise the manuscript. All authors read and approved the final manuscript.
